# Case of a Lumbar Abscess, with an Account of the Appearances on Dissection

**Published:** 1786

**Authors:** Laurence White Maguire

**Affiliations:** Surgeon of the Navy


IV. *Cafe of a Lumbar Abfcefs, with an Account of the Appearances on Diffection. By Mr.* Laurence White Maguire, *Surgeon of the Navy.*

MR. David Lewis, a furgeon of the navy, (and who had been formerly a fellow ftudent with me in London) happening to meet with me here in the month of April laft, requefted quefted me to examine a tumor which his brother, the Rev. Mr. Lewis, had on his loins. It was about three inches in diameter, with a broad bafe, fituated a little on the left fide of the fpine, juft above the pofterior fpinous procefs of the os ileum. It was foft and free from pain, rednefs, or any difcoloration of the fkin.

The patient, who led a ftudious and fedentary life, was about thirty-two years of age, of a thin habit, of a fallow unhealthy complexion, and had been for fome time troubled with a cough. This tumor had made its appearance between three and four months before this period, and he fuppofed that it proceeded from a fall he had received, by which he hurt his back, as it had been gradually increafing from that time, and he had frequently complained of a dull pain in the left thigh, and once thought that he perceived a fmall fwelling in the left groin, but which foon afterwards difappeared: he had alfo fome little degree of lamenefs in walking.

As his general health did not feem to be affected, I concluded, from its appearance, that it was an encyfted tumor of the meliceris kind; but as it feemed to be attached to the aponeurosis of the subjacent muscle, the longissimus dorsi, I thought it would be difficult to separate it from this attachment by dissection; and therefore proposed to make a large incision into the tumor, expecting that such a degree of inflammation would be produced by the incision, as might occasion the cyst to slough away; at least I thought this worth trying; and the idea being approved of by the surgeons who were called in, I proceeded to make a circular incision into the tumor: but when I had penetrated the skin, out gushed a quantity of purulent matter, of a light yellow colour, and rather thin consistence, which left me no room to doubt that it was a lumbar abscess, communicating with the cavity of the abdomen. For this reason, after letting out the matter it contained, (which it should be observed, was much more in quantity than from the apparent size of the tumour could have been expected,) I immediately closed up the wound, and dressed it, giving it as my opinion, that the patient was in great danger. Some other gentlemen present were for enlarging the opening, but as it was the practice in the hospitals that I had attended, to make small openings into these kind of abscesses; and I had the authority of Mr. John Hunter, and

Mr.

Mr. Cline, who in their lectures warn us againſt making large opening in abceſſes communicating with cavities, as they certainly only ſerve to accelerate the patient's death, (general inflammation ſoon after taking place through the whole cavity, the irritation of which brings on violent ſymptoms and death,) I thought myſelf juſtified in not enlarging the wound, and as his brother acquieſced in opinion with me, he was committed to my care.

He grew faint ſoon after the matter was let out; but this ſymptom went off after he was put to bed. The next day, on examination, the wound was found to be united by the adheſive inflammation, and there appeared to be a freſh collection of matter in the tumor, though not ſo large as at firſt. His brother being deſirous to obſerve, whether preſſure on the tumor would communicate any ſenſation to the thigh, placed him with his belly downwards on a table, and preſſed the tumor with his finger; this produced a ſudden and alarming ſyncope, from which he was recovered by being laid on his back on the floor, having the volatile alkali applied to his noſe, &c.

As this ſeemed to be a very dangerous caſe, Mr. Lewis and I thought it beſt to have the opinions of Mr. John Hunter and Mr. Cline concerning it. I therefore drew up a ſtate of the caſe, and ſent it to thoſe gentlemen. Their opinion of the caſe was as unfavourable as ours, and they adviſed little to be done, but rather to wait till the wound ſhould ulcerate again, by which means the matter would be diſcharged without rendering any farther opening neceſſary. This happened, I think, in about three weeks afterwards; and when the wound began to diſcharge afreſh, he was now and then a little faint. The diſcharge now was more thin and ſerous than at firſt, and intermixed with a curdy kind of matter. The next day, about nine o'clock in the morning, he was ſeized with violent pain in the belly, attended with faintneſs and ſickneſs at ſtomach, and had a copious ſtool, after which he had a little eaſe; but the pain returning again with violence, I gave him fifty drops of *tinctura thebaica*, which ſoon relieved him, and the pain returned no more.

The day following he was better, but apt to grow faint on walking about. On introducing a probe into the wound, I could not in any direction make it paſs above an inch, nor could I perceive that when he coughed there was any increaſe of the diſcharge; and yet I was not conconvinced that the ſource of the complaint was not deep ſeated. I now gave him a drachm of bark three times a day, mixed with a little milk, and he continued to take it for ſeveral days afterwards, the quantity being a little increaſed. From the uſe of this remedy his appetite was mended, but the diſcharge continued thin and ſerous, and ſmall in quantity. No bad ſymptoms having come on, I was perſuaded, a few days after it burſt, to enlarge the wound a little; but he could not bear to have it kept open by the lighteſt application of lint, it gave him ſo much uneaſineſs. Some days afterwards, no unfavourable alteration taking place, as the opening was not ſufficiently dependent, and the erect poſition of the patient during the day time, was unfavourable to the free diſcharge of the matter, I ventured to make a counter opening in the lower part of the abceſs, and to introduce a ſeton.

The next day I was obliged to go ſome diſtance into the country for a fortnight; and his brother's buſineſs having forced him to go to town ſome time before, he was obliged to put himſelf under the care of another gentleman. When I returned, I found him very much changed for the worſe, which I was not ſurpriſed at, from from the opinion I had all along entertained of his cafe. He was very much funk, emaciated, and fcarce able to fpeak; and his right thigh and leg were become œdematous to a great degree. From this time he daily grew worfe till he died, which I believe was in lefs than a week after.

His brother was now on the fpot, having returned here on hearing of his being worfe; and confenting to have the body opened; he applied to me to perform that office. I examined the contents of the thorax, where I found no appearance of difeafe, the lungs being found and free from tubercles, nor were there any adhefions of them to the pleura, though he had had a pleuritic complaint feveral months before. The only preternatural appearance in the cheft was a fmall induration on the mediaftinum, between the two lobes of the lungs. The liver and gall bladder were in a natural and healthy ftate, as were likewife the ftomach and inteftines; but behind the peritonæum, over the pfoæ and Iliaci interni mufcles was lodged a furprifing quantity of matter, which was of two confiftencies, the upper part refembling ferum, while the lower part of it was more thick and glairy, and of a pale yellowifh colour; a mixture of both would exactly refenble

ſemble what had flowed out when the tumor was firſt opened; ſo that I imputed this difference of conſiſtence to the ſubſidence of the more ponderous particles of the matter, owing to the body's having lain on its back for ſome time before. The quantity of it could not well be meaſured, but I do not think I exceed, when I ſay there were three quarts of it, lodged in a large ſac of the peritonæum on each ſide of the ſpine, extending from the pelvis conſiderably upwards on each ſide, and raiſing the kidneys from their attachments. Theſe ſacs did not appear to have any communication; for when one of them was opened, and the matter ſuffered to flow out, the other did not collapſe, which muſt have happened, had there been a communication between them.

The pſoæ and iliaci interni muſcles were laid quite bare, and covered with this matter; and on cutting through the right iliacus internus muſcle tranſverſely, there was ſome matter appeared behind it. On the left ſide I traced a communication between the cavity and the external wound, by a ſmall opening between the third and fourth tranſverſe proceſſes of the vertebræ of the loins, which after paſſing through the muſcles of the back, ran obliquely downwards till it reached the external aperture. The right thigh and leg (as I before mentioned) were œdematous to a great degree, and the matter had insinuated itself down among the muscles of the thigh, considerably on each side, but more particularly on the right. The capsular ligament of the joint of the right thigh was in part destroyed, and the head of the os femoris was a little eroded. The only other appearance worth mentioning, was, that the mesenteric glands seemed to be a little enlarged.

*On Board His Majesty's Ship,*

*Standard, at Plymouth,*

Sept. 21, 1785.

